# Probabilistic Risk Assessment of Inorganic Arsenic via Consumption of Herbs Collected in Thailand

**DOI:** 10.1155/2018/8646579

**Published:** 2018-07-15

**Authors:** Nuntawat Monboonpitak, Suthep Ruangwises, Sawanya Buranaphalin, Nongluck Ruangwises

**Affiliations:** ^1^Department of Pharmaceutical Chemistry, Faculty of Pharmacy, Mahidol University, Sri Ayudhya Road, Ratchathewi, Bangkok 10400, Thailand; ^2^Department of Veterinary Public Health, Faculty of Veterinary Science, Chulalongkorn University, Henri Dunant Road, Bangkok 10330, Thailand

## Abstract

Total and inorganic arsenic contents in ten commonly consumed Thai herbs, namely, bird's eye chili, cayenne pepper, celery, garlic, holy basil, kitchen mint, lemongrass, pepper, shallot, and sweet basil, were determined using atomic absorption spectrometry coupled with a hydride generation system (HG-AAS). Total arsenic contents in fresh herbs and lyophilized herbs ranged from 3.39 to 119 ng/g wet weight (wet wt) and from 41.0 to 156 ng/g dry weight (dry wt), respectively. Inorganic arsenic contents in fresh herbs and lyophilized herbs ranged from 2.09 to 26.9 ng/g (wet wt) and from 23.5 to 55.5 ng/g (dry wt), respectively. Percentages of inorganic arsenic to total arsenic in herbs ranged from 22.7 to 62.0%. High percentages of inorganic arsenic to total arsenic were found in celery, lemongrass and sweet basil. Total arsenic contents in the studied herbs were lower than the maximum limits of Thai and Chinese regulatory standards, set at 2,000 ng/g in foods (excluding aquatic animals and seafood) and 500 ng/g in fresh vegetables, respectively. Total and inorganic arsenic contents in the studied herbs were comparable to or lower than the levels found in other studies in the EU and China. Lifetime average daily dose (LADD) and cancer risk (CR) of inorganic arsenic exposure to commonly consumed herbs were evaluated using probabilistic risk assessment (PRA) by @RISK software version 6.0 of Palisade cooperation. All calculated LADD and CR values from all herbs did not exceed the acceptable levels. It can be concluded that there were very low cancer risks of inorganic arsenic exposure from the consumption of the studied herbs.

## 1. Introduction

Herbs have played an important role in traditional medicines and foods for centuries. At present, approximately 4 billion people (representing 80% of the world's population), especially in developing countries, use herbs or medicinal plants for therapeutic purposes or primary healthcare [1]. Traditional medicines produced from herbs or medicinal plants are used worldwide, with an increasing interest in developed or Western countries [2]. The increased application has raised concerns about the potential adverse effects of these herbs. In comparison to synthetic drugs, herbal drugs are generally considered to have a lower risk of side effects; however, they could possibly contain some toxic substances, such as heavy metals [3].

Arsenic (As) is a widespread environmental contaminant, resulting from both natural occurrences and human activities [4, 5]. Volcanic eruptions and other natural processes are sources of arsenic in the environment. Human activities, including disposal of industrial chemicals, smelting of arsenic-bearing minerals, burning of fossil fuels, and the application of arsenic compounds in numerous products, also cause arsenic contamination [4, 5]. Arsenic compounds are used in many manufacturing processes, including glass, electrical devices, pesticides, and pigments [5]. In addition, arsenic-containing pesticides that were widely used in the past have caused some agricultural areas to become contaminated with arsenic [6].

Acute and chronic toxicity of arsenic can involve the respiratory, cardiovascular, nervous and hematopoietic systems [4]. Inorganic arsenic compounds are more toxic than organic compounds, and the trivalent forms are more harmful than the pentavalent forms [5]. Inorganic arsenic compounds have been identified as human carcinogens, with evidence for an increased cancer risk of the urinary bladder, lung, and skin. Moreover, long-term exposure to inorganic arsenic has been reported to be associated with skin lesions, mental disorders, cardiovascular diseases, neurotoxicity, and diabetes [5]. The International Agency for Research on Cancer (IARC) has classified inorganic arsenic compounds as Group 1 carcinogens [7]. In 2010, the provisional tolerable weekly intake (PTWI) of arsenic at 15 *μ*g/kg body weight (bw)/week was withdrawn. The Joint FAO/WHO Expert Committee on Food Additives (JECFA) has determined a benchmark dose lower confidence limit for a 0.5% increased incidence of lung cancer (BMDL_0.5_) of 3.0 *μ*g/kg bw/day (ranging from 2.0 to 7.0 *μ*g/kg bw/day) [8]. European Food Safety Authority has identified a range of benchmark dose lower confidence limit (BMDL01) values of 0.3 and 8 *μ*g/kg bw/day for cancers of the lung, skin, and bladder [9].

Foods are major sources of arsenic exposure in the general population. The dietary exposure to arsenic varies widely, depending on the food type, cultivation practices, environmental factors, and food processing methods [5]. The consumption of herbs can contribute to arsenic exposure because of arsenic contamination in the environment in which plants are cultivated (soil, irrigation water, and the atmosphere). Little information has been reported on the arsenic levels in Thai herbs. Thus, the aim of this study was to determine the total and inorganic arsenic contents in ten commonly consumed Thai herbs and to evaluate the health risks of inorganic arsenic exposure to commonly consumed herbs by the Thai population.

## 2. Materials and Methods

### 2.1. Chemicals

Nitric acid (HNO_3_) and hydrochloric acid (HCl) were purchased from Merck (Darmstadt, Germany). Dimethylarsinic acid (DMA), hydrazine sulfate, hydrobromic acid, and other chemicals were purchased from Sigma-Aldrich (St. Louis, MO, USA). Rice flour (standard reference material [SRM] 1568a) was obtained from the National Institute of Standards and Technology (Gaithersburg, MD, USA). All standard solutions, reagents, and samples were prepared using deionized water (18 MΩcm). To avoid arsenic residue contamination, all glassware was soaked in 10% (v/v) HNO_3_ overnight and washed three times with deionized water before use.

### 2.2. Sample Collection

Ten herbs commonly consumed in Thailand were used in this study (Table 1). The samples were collected between February and May 2015 from 10 provinces (Chiang Mai, Chiang Rai, Khon Kaen, Lampang, Nakhon Pathom, Nakhon Ratchasima, Songkla, Suphan Buri, Trang, and Tak) of Thailand. Approximately 500 g of each sample was purchased from local markets and stored in clean plastic bags.

### 2.3. Sample Preparation

A total of 150 samples (15 samples for each herb) were determined for total and inorganic arsenic. The samples were first washed through tap water. Only the edible parts of each herb sample were used. The cleaned edible parts of samples were cut and air-dried, then frozen, and lyophilized. Each lyophilized sample was ground into fine powder using a mortar and pestle. Each powdered sample was passed through a fine mesh sieve and stored in an airtight container at 4°C until analysis.

### 2.4. Determination of Total Arsenic

Determination of total arsenic was performed using the method described by Muñoz et al. [12]. An aliquot of lyophilized sample (0.250 g) was mixed with 1 mL of ashing suspension (20% w/v Mg(NO_3_)_2_·6H_2_O and 2% w/v MgO in water) and 5 mL of 50% (v/v) HNO_3_. The mixture was evaporated on a hot plate to dryness and then mineralized at 450°C in a furnace. The resulting white ash was dissolved in 5 mL of 6 N HCl and 5 mL of a reducing solution (5% w/v KI and 5% w/v ascorbic acid). The solution was left for 30 min and then 10 mL of 50% v/v HCl was added to the solution. The solution was filtered through a Whatman No. 1 filter paper into a 25 mL volumetric flask and adjusted to volume with 50% v/v HCl. The resulting solution was used for determination of total arsenic. All samples were analyzed in duplicate.

### 2.5. Determination of Inorganic Arsenic

Inorganic arsenic was determined by the method described by Muñoz et al. [12]. An accurate weight (0.500 g) of lyophilized sample was placed in a 50 mL screw-capped centrifuge tube; 4.1 mL of water was added to the sample and mixed until completely moistened. In order to hydrolyze As(III) from the thiol groups of proteins, 18.4 mL of concentrated HCl was added to the moistened sample, shaken for 1 h, and left overnight (12–15 h). Reducing agent (1 mL of 1.5% [w/v] hydrazine sulfate and 2 mL of hydrobromic acid) was added to the sample tube and vortexed for 2 min. Chloroform (10 mL) was added to the tube, which was then shaken and centrifuged at 1,000 ×* g* for 10 min using a bench-top centrifuge (model 5810; Eppendorf, Hamburg, Germany). The chloroform phase was aspirated into another centrifuge tube. The extraction process was repeated twice. The chloroform phase was then filtered through a syringe filter with a 25 mm polytetrafluoroethylene membrane, pore size 0.5 *μ*m (ChromTech, Apple Valley, MN, USA) into another 50-mL tube. Inorganic arsenic in the chloroform phase was extracted with 10 mL of 1 N HCl and centrifuged (1,000 ×* g* for 10 min). The aqueous phase was aspirated into a beaker. The extraction process was repeated once more. The amount of inorganic arsenic in the combined aqueous acid phase was quantified as described in the determination of total arsenic, with the addition of 2.5 mL ashing suspension and 10 mL of 50% (v/v) HNO_3_. All samples were analyzed in duplicate.

### 2.6. Instrumentation

An atomic absorption spectrophotometer (A Analyst 300; PerkinElmer, Waltham, MA, USA), equipped with an AS-90 autosampler, FIAS 400 flow injection system, arsenic hollow-cathode lamp, and hydride generation, was used for determination of total and inorganic arsenic contents in the final solutions. The operating conditions for HG-AAS were as described by Ruangwises et al. (2012) [13].

### 2.7. Determination of Limits of Quantitation

Limits of quantitation (LOQ) for total and inorganic arsenic were determined using the Q2B method of the USFDA [14]. For determination of the LOQ for total arsenic, lyophilized plant samples (0.250 g) were fortified with an arsenic mixture [As(III): As(V) 1:1 w/w] at concentrations of 250, 500, 1,000 and 2,500 ng/g; blank samples were not fortified with arsenic. Concentrations of total arsenic in fortified and blank samples were quantified as described in the determination of total arsenic. For determination of the LOQ for inorganic arsenic, lyophilized plant samples (0.500 g) were fortified with an arsenic mixture at concentrations of 50, 100, 500, and 1,000 ng/g; blank samples were not fortified with arsenic. Concentrations of inorganic arsenic in fortified and blank samples were quantified as described in the determination of inorganic arsenic.

### 2.8. Quality Assurance

The accuracy of total and inorganic arsenic determinations was assessed by determining the total and inorganic arsenic contents in the standard reference material (SRM) 1568a (rice flour). The accuracy was also tested using different concentrations of fortified samples, calculated as % recovery. Intraday and interday precision were expressed as percentage of relative standard deviation (% RSD).

### 2.9. Statistical Analysis

Statistical tests were conducted using the SPSS Statistics software program, version 18. One-way analysis of variance (ANOVA) with Tukey's post hoc test (*p* < 0.05) was used to examine the differences of total and inorganic arsenic contents in different kinds of herbs.

### 2.10. Intake Rate of Herbs

Individual intake rate of the studied herbs were obtained from the National Bureau of Agricultural Commodity and Food Standards with permission. The food consumption data of Thailand was conducted during 2003–2004. This consumption survey used food frequency questionnaires. The number of subjects included in the study was 18,746 people (9,316 male and 9,430 female; 2,363 of age < 3 years and 16,383 of age ≥ 3 years) from 17 provinces in Thailand including Bangkok. Intake rate data was used to calculate deterministic risk assessment and probabilistic risk assessment. Not all intake rate of the studied herbs has been reported; therefore the intake rate of some herbs was justified based on the available data.

### 2.11. Lifetime Average Daily Dose (LADD) and Cancer Risk (CR)

Lifetime average daily dose and cancer risk of inorganic arsenic exposure to commonly consumed herbs were evaluated using probabilistic risk assessment (PRA). PRA has been used in exposure assessment to estimate lifetime average daily exposure concentration by considering human variation and uncertainty [15]. PRA used @RISK software version 5.5 of Palisade cooperation. The probabilistic assessment was conducted by Monte Carlo simulation which estimates the possibility of the incidence. The appropriate distribution was selected to fit with the data. Each variable from the particular distribution of both arsenic concentration data and intake rate of herb were randomized using 10,000 iterations. Two types of exposure assessment were used including central tendency estimate (CTE) and reasonable maximum estimate (RME). The CTE model was calculated from average inorganic concentration and intake rate of the particular herb. The RME model was calculated from the 95th percentile of inorganic concentration and intake rate of the particular herb.

Lifetime average daily doses (LADD) from the consumption of the studied herbs were estimated by the following equation:

(1) $$\begin{array}{c} LADD=\frac{C\times IR\times ED}{BW\times LT} \end{array}$$

where LADD is lifetime average daily doses (mg/kg/day); C is concentration of chemical (mg/kg food); IR is intake rate (kg/day); BW is body weight (kg); ED is exposure duration (years); LT is lifetime (days).

Cancer risk characterization of inorganic arsenic exposure was estimated using the following equation:

(2) $$\begin{array}{c} CR=LADD\times CSF_{o} \end{array}$$

where CR is cancer risk, a unitless probability; CSF_o_ is oral cancer slope factor for inorganic arsenic =1.5 per mg/kg bw/day.

## 3. Results

The accuracy of this analytical method was assessed by determination of the total arsenic and inorganic arsenic contents in SRM 1568a rice flour. Concentrations of total arsenic and inorganic arsenic found in rice flour were 283 ± 34 ng/g (*n* = 10, reference value of 290 ± 30 ng/g) and 102 ± 4 ng/g (*n* = 10), respectively [13]. The concentration of inorganic arsenic was in agreement with previous reports of 111 ± 6 ng/g [16] and 111 ± 3 ng/g [17] using the same method of analysis.

The accuracy using different concentrations of fortified samples, calculated as % recovery, was also tested. The intraday and interday precision was expressed as percentage of relative standard deviation (% RSD). The % recovery and % RSD of the total and inorganic arsenic determinations in herb samples fortified with arsenic mixture at four concentrations were in an acceptable range (Table 2). The average recoveries across the four concentrations of fortified arsenic mixtures were 95.6% and 95.4% for total and inorganic arsenic, respectively. The precision of the method was calculated with the equation % RSD = 100SD/$\overset{-}{x}$, where SD is the standard deviation and $\overset{-}{x}$ is the mean arsenic concentration recovered from the arsenic-fortified samples. The % RSD ranged from 1.70 to 5.00% for total arsenic and from 1.18 to 5.38% for inorganic arsenic. The calculation for LOQ was based on the standard deviation of* y*-intercepts from linear regression analysis (ó) and the mean of the slope (S), using the equation LOQ = 10 ó/S. The LOQs for total and inorganic arsenic in herb samples were 19 and 15 ng/g dry weight, respectively. Since different moisture contents were found in each herb, LOQs for total and inorganic arsenic (wet wt) were calculated for individual herbs (Table 3).

Total and inorganic arsenic contents and the percentage of inorganic arsenic to total arsenic in ten herbs collected in Thailand are shown in Table 4. There was a wide variation of total arsenic content in the studied herbs, ranging from 3.39 to 119 ng/g wet weight (wt), with a significant difference (*p* < 0.05). The highest level of total arsenic was found in pepper (119 ng/g wet wt). Total arsenic content in lyophilized herbs ranged from 41.0 to 156 ng/g dry wt, with a significant difference (*p* < 0.05). The highest contents of total arsenic (dry wt) were found in shallot (156 ng/g), pepper (132 ng/g) and holy basil (128 ng/g).

Inorganic arsenic content in herbs ranged from 2.09 to 26.9 ng/g wet wt, with a significant difference (*p* < 0.05). Pepper also contained the highest level of inorganic arsenic (26.9 ng/g wet wt). Inorganic arsenic content in lyophilized herbs ranged from 23.5 to 55.5 ng/g dry wt, with a significant difference (*p* < 0.05). The highest contents of inorganic arsenic (dry wt) were found in lemongrass (55.5 ng/g), holy basil (46.8 ng/g) and sweet basil (46.3 ng/g). Percentages of inorganic arsenic to total arsenic in herbs ranged from 22.7 to 62.0%, with the highest percentages occurring in celery (62.0%), lemongrass (56.0%), sweet basil (52.2%) and cayenne pepper (47.7%).

The intake rates of the studied herbs are presented in Table 5. For the average intake level, the highest intake was found in garlic (3.36 g/day) followed by lemongrass (2.87 g/day) and shallot (2.68 g/day), while, at the 95 percentile intake level, the highest intake was found in lemongrass (13.4 g/day), garlic (10.0 g/day), and bird's eye chili and cayenne pepper (9.6 g/day).

Table 6 shows lifetime average daily dose (LADD) and cancer risk (CR) of inorganic arsenic exposure from the studied herbs consumption using PRA with CTE and RME models. Cancer risk is the theoretical maximum number of cancer cases that are expected to develop due to the exposure to a carcinogen. The acceptable lifetime cancer risk for inorganic arsenic is 10^−5^ (1 in 100,000). The highest LADD and CR of inorganic arsenic exposure were found from the consumption of lemongrass. All LADD values calculated from both CTE and RME models of inorganic arsenic exposure from all herbs were much lower than the benchmark dose level (BMDL_0.5_) for lung cancer established by JECFA at 3 × 10^−3^ mg/kg/day (2–7 × 10^−3^ mg/kg/day based on the range of estimated total dietary exposure). Cancer risk values calculated from the RME model of inorganic arsenic exposure from all herbs do not exceed the acceptable cancer risk at 10^−5^. The results indicated that there were very low cancer risks of inorganic arsenic exposure from consumption of the studied herbs.

## 4. Discussion

Established maximum levels for total and inorganic arsenic in herbs are scarce. Thailand has set a maximum level of 2,000 ng/g total arsenic in foods (excluding aquatic animals and seafood) [18], while China has established a maximum limit of 500 ng/g total arsenic in fresh vegetables [19]. Total arsenic contents in the studied herbs were lower than the Thai and Chinese regulatory standards. Considering rice which is a staple food contributing to high intake of inorganic arsenic exposure [8], maximum levels for inorganic arsenic contents in husked rice and polished rice were determined by Codex at the levels of 350 and 200 ng/g, respectively [20], while European Union (EU) has established maximum levels of inorganic arsenic contents in husked rice and polished rice of 250 and 200 ng/g, respectively [21]. Maximum level of inorganic arsenic in rice of 200 ng/g has also been set by the Chinese government [19]. Inorganic arsenic contents in all herbs in the present study were also below the maximum levels of inorganic arsenic in rice established by Codex, EU and China.

Studies on the total and inorganic arsenic contents in herbs have been limited. As shown in Table 7, total and inorganic arsenic contents in Thai herbs from the present study were comparable to or lower than the levels found in other studies [10, 11]. The total and inorganic arsenic contents in dietary supplements (based on herbs, other botanicals and algae) sold in Denmark were reported in the range of 580–5000 and 30–3200 ng/g, respectively [22] which were much higher than the levels of total and inorganic arsenic found in the present study. It could be due the fact that to the diet supplements were prepared from the extract or dry powder of plant materials. In addition, the major herbal or plant ingredients of the dietary supplements studied [22] were different from the herbs used in our study.

Cancer risks of inorganic arsenic from herb consumption in this study were lower than the previous studies. A few studies reported cancer risk of inorganic arsenic from herb or vegetable consumption. Rehman et al. (2016) reported CR of inorganic arsenic from different vegetable consumption in Pakistani population ranged from 4.67 × 10^−6^ (winter melon) to 98.8 × 10^−6^ (coriander) [23]. Uddh-Söderberg et al. (2015) reported the CR at 2 x 10^−4^ of arsenic exposure via consumption of homegrown vegetables near contaminated glassworks sites in Sweden [24].

This study shows that there were very low cancer risks of inorganic arsenic exposure from consumption of the studied herbs. Further research work may integrate the inorganic arsenic exposure from herb consumption in this study with other dietary sources to investigate the cancer risk from dietary exposure to inorganic arsenic in Thai population.

## 5. Conclusions

The present findings show that arsenic contents in the studied herbs collected in Thailand were lower than the available maximum limits for arsenic that have been established for various commodities. It can be concluded that, in terms of arsenic levels, these Thai herbs are safe for consumption and exportation. However, regular monitoring of inorganic arsenic contamination in plants and risk assessment of inorganic arsenic exposure are suggested to ensure food safety.

## Figures and Tables

**Table 1:** Characteristics of the studied Thai herbs.

Common name	Scientific name	Family	Edible part
Bird's eye chili	*Capsicum frutescens*	Solanaceae	Fruits
Cayenne pepper	*Capsicum annuum *	Solanaceae	Fruits
Celery	*Apium graveolens *	Apiaceae	Leaves/Stems
Garlic	*Allium sativum *	Alliaceae	Clove
Holy basil	*Ocimum sanctum*	Lamiaceae	Leaves
Kitchen mint	*Mentha cordifolia*	Lamiaceae	Leaves
Lemongrass	*Cymbopogon citratus*	Gramineae	Stems
Pepper	*Piper nigrum*	Piperaceae	Fruits
Shallot	*Allium cepa*	Alliaceae	Clove
Sweet basil	*Ocimum basilicum*	Lamiaceae	Leaves

**Table 2:** Accuracy (% recovery) and precision (% RSD) in determination of total and inorganic arsenic in herbs.

Arsenic spiked (ng/g)	Intraday (*n* = 6)			Interday (*n* = 6)		
	Found (ng/g) (mean ± SD)	RSD (%)	Recovery (%)	Found (ng/g) (mean ± SD)	RSD (%)	Recovery (%)
Total arsenic						
250	244 ± 8.53	3.49	97.7	237 ± 6.68	2.82	94.9
500	473 ± 13.6	2.88	94.6	478 ± 21.2	4.43	95.6
1000	945 ± 32.6	3.45	94.5	972 ± 48.5	5.00	97.2
2500	2,370 ± 41.4	1.75	94.7	2.38 × 10^3^ ± 40.5	1.70	95.3
Inorganic arsenic						
50	47.4 ± 2.54	5.37	94.8	47.7 ± 2.57	5.38	95.3
100	98.1 ± 4.81	4.91	98.1	97.9 ± 3.36	3.43	97.9
500	469.8 ± 25.2	5.35	94.0	473 ± 21.9	4.63	94.6
1000	943.8 ± 13.4	1.42	94.4	941 ± 11.1	1.18	94.1

**Table 3:** Moisture contents and limits of quantification (LOQs) of total and inorganic arsenic of individual herbs.

	Average Moisture Content (%)	LOQ (total arsenic wet wt)	LOQ (inorganic arsenic, wet wt)
Bird's eye chili *(Capsicum frutescens)*	64.2	6.80	5.37
Cayenne pepper *(Capsicum annuum)*	73.7	5.00	3.95
Celery *(Apium graveolens)*	91.6	1.60	1.26
Garlic *(Allium sativum)*	86.5	2.57	2.03
Holy basil *(Ocimum sanctum)*	79.5	3.90	3.08
Kitchen mint *(Mentha cordifolia)*	87.0	2.47	1.95
Lemongrass *(Cymbopogon citratus)*	68.2	6.04	4.77
Pepper *(Piper nigrum)*	9.53	17.2	13.6
Shallot *(Allium cepa)*	91.9	1.54	1.22
Sweet basil *(Ocimum basilicum)*	81.6	3.50	2.76

**Table 4:** Total and inorganic arsenic contents and percentage of inorganic arsenic in Thai herbs, expressed as mean ± standard deviation (minimum–maximum).

Herb	Total arsenic (ng/g)		Inorganic arsenic (ng/g)		% Inorganic arsenic^*∗*^
	Wet weight	Dry weight	Wet weight	Dry weight	
Bird's eye chili *(Capsicum frutescens)*	40.5 ± 14.9^b^ (28.0–79.2)	113 ± 39.7^b^ (78.3–219)	14.4 ± 3.72^c^ (9.31–23.5)	40.2 ± 10.2^bc^ (24.9–63.8)	36.6 ± 5.95^ce^ (27.2–46.1)
Cayenne pepper *(Capsicum annuum)*	16.4 ± 2.21^ef^ (13.3–19.9)	62.7 ± 8.26^de^ (47.2–81.9)	7.83 ± 1.74^d^ (6.15–13.3)	29.9 ± 7.31^ce^ (23.8–54.6)	47.7 ± 7.39^ac^ (35.7–66.7)
Celery *(Apium graveolens)*	3.39 ± 0.940^g^ (1.96–5.81)	41.0 ± 11.8^e^ (30.4–77.5)	2.09 ± 0.566^e^ (1.28–3.37)	25.3 ±6.81^de^ (17.2–39.6)	62.1 ± 7.93^a^ (48.8–75.4)
Garlic *(Allium sativum)*	8.01 ± 2.06^fg^ (6.49–13.1)	59.9 ± 12.8^de^ (42.6–79.8)	3.15 ± 1.00^e^ (/2.05–5.54)	23.5 ± 6.60^e^ (15.5–36.9)	39.3 ± 6.46^bc^ (30.7–48.8)
Holy basil *(Ocimum sanctum)*	26.6 ± 7.13^cd^ (13.2–44.9)	128 ± 22.8^ab^ (73.4–177)	9.64 ± 2.61^d^ (5.95–14.6)	46.8 ± 9.97^ab^ (31.4–66.1)	37.0 ± 7.19^ce^ (24.8–47.0)
Kitchen mint *(Mentha cordifolia)*	10.5 ± 3.00^eg^ (6.46–16.0)	80.6 ± 16.1^cd^ (49.0–96.8)	4.00 ± 1.08^e^ (1.95–5.77)	31.0 ± 7.20^ce^ (16.6–42.8)	38.6 ± 5.65^bcd^ (30.2–46.7)
Lemongrass *(Cymbopogon citratus)*	32.0 ± 5.38^bc^ (23.4–43.3)	100 ± 16.1^bc^ (73.4–127)	17.7 ± 2.81^b^ (14.2–25.2)	55.5 ± 8.12^a^ (44.5–73.8)	56.0 ± 8.43^a^ (44.4–68.3)
Pepper *(Piper nigrum)*	119 ± 19.0^a^ (84.2–149.6)	132 ± 21.0^ab^ (92.4–166)	26.9 ± 5.39^a^ (13.9–33.5)	29.7 ± 6.03^ce^ (15.3–37.3)	22.7 ± 4.30^e^ (15.4–31.1)
Shallot *(Allium cepa)*	12.6 ± 5.05^eg^ (5.13–26.3)	156 ± 52.2^a^ (87.0–263)	2.87 ± 1.07^e^ (1.34–4.86)	34.6 ± 7.42^cd^ (22.7–50.1)	24.1 ± 7.15^de^ (10.5–32.5)
Sweet basil *(Ocimum basilicum)*	18.6 ± 6.09^de^ (10.0–35.4)	102 ± 37.7^bc^ (59.2–207)	8.56 ± 3.40^d^ (6.33–20.3)	46.3 ± 16.6^ab^ (30.8–104)	52.2 ± 33.7^ab^ (22.3–159)

A total of 150 samples (15 samples for each herb) were determined for total arsenic and inorganic arsenic.

^a,b,c,d,e,f,g^ = mean values with different superscripts in the same column are significantly different (*p* < 0.05).

*∗* % inorganic arsenic = (concentration of inorganic arsenic × 100)/concentration of total arsenic.

**Table 5:** Intake rates of the studied herbs.

Herb	Intake rate*∗*	
	Average intake (g/day)	95 Percentile intake (g/day)
Bird's eye chili	1.49	9.60
Cayenne pepper	1.49	9.60
Celery	0.94	3.75
Garlic	3.36	10.0
Holy basil	0.18	1.00
Kitchen mint	0.94	3.75
Lemongrass	2.87	13.4
Pepper	0.13	0.50
Shallot	2.68	9.00
Sweet basil	0.18	1.00

*∗*Probability distribution of intake rate was conducted by Monte Carlo simulation using @RISK software version 5.5 of Palisade cooperation.

**Table 6:** LADD and CR of inorganic arsenic exposure from the studied herbs using probabilistic risk assessment.

Herb	LADD (mg/kg bw/day)		CR	
	CTE	RME	CTE	RME
Bird's eye chili	4.01 x 10^−7^	12.7 x 10^−7^	6.02 x 10^−7^	19.1 x 10^−7^
Cayenne pepper	2.13 x 10^−7^	6.59 x 10^−7^	3.20 x 10^−7^	9.89 x 10^−7^
Celery	0.37 x 10^−7^	1.20 x 10^−7^	0.56 x 10^−7^	1.79 x 10^−7^
Garlic	2.78 x 10^−7^	9.26 x 10^−7^	4.17 x 10^−7^	13.9 x 10^−7^
Holy basil	0.32 x 10^−7^	1.00 x 10^−7^	0.48 x 10^−7^	1.50 x 10^−7^
Kitchen mint	0.71 x 10^−7^	2.30 x 10^−7^	1.06 x 10^−7^	3.45 x 10^−7^
Lemongrass	9.40 x 10^−7^	29.0 x 10^−7^	14.1 x 10^−7^	43.6 x 10^−7^
Pepper	0.620 x 10^−7^	1.94 x 10^−7^	0.920 x 10^−7^	2.91 x 10^−7^
Shallot	1.46 x 10^−7^	4.82 x 10^−7^	2.19 x 10^−7^	7.22 x 10^−7^
Sweet basil	0.270 x 10^−7^	0.840 x 10^−7^	0.400 x 10^−7^	1.26 x 10^−7^

**Table 7:** Total and inorganic arsenic contents in some herbs from the present study compared to the available literature data.

Herb	Location	Total arsenic (ng/g wet wt)	Inorganic arsenic (ng/g wet wt)	Reference
Bird's eye chili *(Capsicum frutescens)*	EU	–	9.00	[10]
	Thailand	40.5	14.4	Present study
Cayenne pepper (*Capsicum annuum*)	EU	–	12.0	[10]
	Thailand	16.4	7.80	Present study
Celery (*Apium graveolens*)	EU	–	10.9	[10]
	China	72.1	–	[11]
	Thailand	3.40	2.90	Present study
Garlic (*Allium sativum*)	China	60.2	–	[11]
	Thailand	8.00	3.20	Present study
Pepper (*Piper nigrum*)	EU	–	53.6	[10]
	Thailand	119	26.9	Present study
Sweet basil (*Ocimum basilicum*)	EU	–	31.6	[10]
	Thailand	18.6	8.60	Present study

– = no data.
